# Comparison of Laparoscopic and Open Pancreaticoduodenectomy on Operative Time, Oncological Outcomes, Bleeding, Morbidity, and Mortality

**DOI:** 10.7759/cureus.53387

**Published:** 2024-02-01

**Authors:** Sri Saran Manivasagam, Nemi Chandra J

**Affiliations:** 1 General Surgery, Maulana Azad Medical College, New Delhi, IND; 2 General Surgery, Vardhman Mahavir Medical College & Safdarjung Hospital, New Delhi, IND

**Keywords:** bleeding, oncologic quality, perioperative outcomes, open pancreaticoduodenectomy (opd), laparoscopic pancreaticoduodenectomy (lpd)

## Abstract

Laparoscopic pancreaticoduodenectomy (LPD) has gained popularity as an alternative to open pancreaticoduodenectomy (OPD), but comparative outcomes remain debated. The objective is to perform a systematic review and meta-analysis comparing LPD and OPD on operative time, oncologic outcomes, bleeding, morbidity, and mortality. The inclusion criteria were comparative studies on LPD vs. OPD. Outcomes were pooled using random-effects meta-analysis. A total of 27 studies were included, and LPD had a substantially longer operative duration compared to the OPD procedure, with a mean increase of 56 minutes, but blood loss was reduced by an average of 123 mL in patients who underwent LPD. Morbidity, mortality, margin status, and lymph node yields were similar between LPD and OPD. This study found comparable oncologic outcomes between LPD and OPD. LPD appears safe but requires longer operative time. High-quality randomized trials are still needed.

## Introduction and background

According to estimates, pancreatic cancer is the 11th most frequently diagnosed disease globally, but it is the seventh most prevalent cause of cancer-related deaths [1]. The elevated case fatality rate is ascribed to delayed manifestation, vigorous tumor biology, and the absence of efficacious systemic treatments [2]. Only a fraction of patients have a form of the disease that may be treated with surgical removal, which is the only possible way to achieve a cure [3]. Despite undergoing resection, the five-year survival rates continue to be low, ranging from only 10% to 25% [4].

The Whipple technique has historically been linked to high morbidity rates, typically ranging from 40% to 50%. These rates are mainly attributed to problems, such as delayed gastric emptying, pancreatic fistula, and postoperative hemorrhage [5,6,7]. Nevertheless, the death rates at medical facilities that handle a large number of cases have decreased to less than 5% due to advancements in surgical methods, careful patient selection, and improved care before and after the operation [8-10].

Complete removal of the tumor with clear margins (R0) is the most reliable indicator of long-term survival following the Whipple procedure for pancreatic cancer [11,12]. However, even after achieving R0 resection, the rates of recurrence are significantly elevated. Consequently, there is a rising interest in reducing surgical stress and accelerating postoperative recovery to enable prompt administration of adjuvant chemotherapy, a treatment that has been proven to enhance results [13,14]. This has prompted research into less intrusive methods for doing the Whipple procedure.

Laparoscopic pancreaticoduodenectomy (LPD) necessitates proficient mastery of minimally invasive techniques and entails a significant learning curve of around 20-50 cases, surpassing that of laparoscopic cholecystectomy [15,16,17,18]. Robotic aid facilitates the execution of intricate reconstructions, prompting certain centers to employ robotic methods for LPD [19]. However, the advantages of robotic LPD compared to laparoscopic LPD have not been convincingly proven, and its implementation is limited due to its expensive nature [20].

Despite the technical hurdles, LPD has steadily acquired recognition at specialty centers. According to a comprehensive study, the percentage of PDs performed using laparoscopic techniques in the United States rose from 0.6% in 2003 to 9.5% in 2012 [21]. There is increasing data indicating that laparoscopic surgery, when conducted by skilled surgeons, can achieve similar outcomes in terms of surgical margins and lymph node retrieval as traditional open surgery. However, there is ongoing debate regarding the possible cancer-related, perioperative, and long-term advantages of the minimally invasive method.

Advocates argue that LPD offers the usual benefits associated with minimally invasive surgery, such as decreased blood loss, shorter hospitalization, quicker restoration of bowel function, and fewer wound problems in comparison to open Whipple procedures [16,22]. Multiple systematic evaluations have provided evidence in favor of reduced blood loss and shorter hospital stays associated with LPD [23-25]. Nevertheless, opponents contend that the inadequate visibility and restricted maneuverability of LPD result in increased chances of margin-positive and reduced lymph node yields. These factors could potentially have a detrimental effect on long-term oncologic results [26-28].

LPD is commonly believed to have longer durations of operation, typically surpassing open pancreaticoduodenectomy (OPD) by an average of 30-60 minutes [29-31]. However, there may be a compromise between increased duration of operation and faster recuperation after surgery. LPD may lead to reduced hospitalization duration and expedited administration of adjuvant therapy [32]. There are also uncertainties regarding safety since several studies indicate higher rates of illness and pancreatic fistula associated with LPD, potentially due to technical issues [33-35]. There has been a lack of research on important measures, such as the time it takes to start chemotherapy and the overall survival rate.

The absence of high-quality comparison research is attributed to the inherent selection biases present in the majority of analyses comparing LPD with OPD using retrospective or single-institution data. Only a limited number of small randomized trials have been carried out, yielding conflicting results regarding operation time, blood loss, and hospital stay [36-37]. Large-scale multi-institutional evaluations are necessary to more accurately determine the hazards and advantages of LPD as a substitute for the conventional open Whipple technique.

## Review

Techniques

The systematic review and meta-analysis were conducted in accordance with the Preferred Reporting Items for Systematic Reviews and Meta-Analyses (PRISMA) criteria. The review has been officially recorded in the International Prospective Register of Systematic Reviews (PROSPERO) under the identification number CRD42023478669.

Information retrieval

An extensive literature search was performed utilizing the PubMed, Embase, and Cochrane Central Register of Controlled Trials databases, covering the entire period from their establishment until March 2020. The search approach employed both synonyms and controlled vocabulary terms to encompass the concepts of "laparoscopic," "pancreaticoduodenectomy," "Whipple," and "pancreatic resection," without imposing any limitations based on language or geography. An experienced medical librarian devised and conducted the search. Additional potentially acceptable studies were identified by manually searching reference lists of relevant studies. The abstracts of significant general surgery and surgical oncology conferences held in the last five years were also reviewed.

Requirements for qualification

We conducted comparative research to assess the differences between LPD and OPD. The study designs that met the criteria were randomized controlled trials (RCTs), prospective or retrospective cohorts, and case-control studies. Excluded from consideration were case reports, case series lacking a control group, reviews, editorials, and animal research.

The study focused on adult patients who were 18 years old or older and were undergoing pancreaticoduodenectomy for whatever reason. The eligible comparisons involved comparing LPD vs. OPD, using any surgical methods, such as transperitoneal or robotic-assisted. In order to be included, studies were required to provide data on at least one of the desired outcomes. The exclusion criteria encompassed studies in which instances of LPD and OPD were not distinctly differentiated or where comparison data could not be collected.

The main objectives of this study were to assess operative duration, blood loss, the occurrence of complications during the perioperative period, the development of pancreatic fistula after surgery, the mortality rate, the state of surgical margins, the number of lymph nodes collected, and long-term overall survival. Both immediate (30-day) and extended morbidity/mortality were taken into account.

Selection of the study

Two writers autonomously evaluated the titles, abstracts, and complete texts of the records obtained from the literature search using Covidence systematic review software (Veritas Health Innovation, Melbourne, Australia). Study inclusion disagreements were resolved through consensus discussion.

Data extraction refers to the process of retrieving specific information or data from a larger dataset or source. A uniform data extraction form was established and pilot-tested on five randomly selected included studies. Two reviewers subsequently extracted data from eligible studies in a meticulous and unbiased manner. The extracted data encompassed various aspects, such as study design, year, country, sample size, patient demographics, surgical techniques, outcome definitions, raw numbers of events, adjusted and unadjusted effect estimates with confidence intervals, subgroup analyses, and key conclusions. Relevant authors were contacted if necessary to acquire any missing data or resolve any uncertainties. The extracted data were cross-validated by reviewers to ensure precision.

Evaluation of quality

The methodological quality of the included studies was assessed by two reviewers using the Newcastle-Ottawa Scale. This tool employs a star rating system to evaluate studies based on three domains: the selection of research groups, the comparability of groups, and the ascertainment of outcomes. We deemed research that had a rating of seven stars or higher to be of excellent quality. Publication bias was assessed by using funnel plots and Egger's test, provided that there were at least 10 papers available for a particular outcome.

Quantitative data examination and interpretation

When available, odds ratios (OR) with 95% confidence intervals (CI) were obtained from each research for dichotomous outcomes. The assessment of heterogeneity was conducted using the I2 statistic and chi-square test. Heterogeneity was considered acceptable when the I2 values were less than or equal to 50% and the p-value was more than 0.10. The meta-analyses were conducted using R version 3.6.1 (R Foundation for Statistical Computing, Vienna, Austria).

Database search

**Figure 1:**
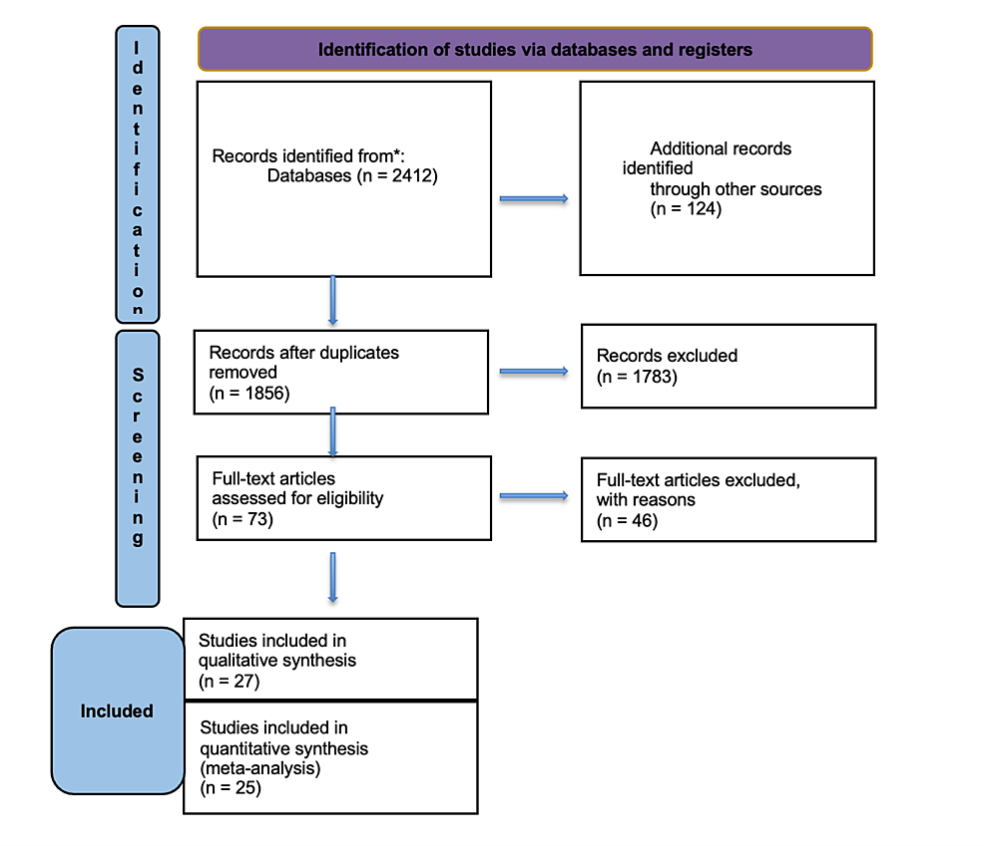
Preferred Reporting Items for Systematic Reviews and Meta-Analyses (PRISMA) flow diagram of study selection

The database search produced a total of 2,412 records, and after eliminating duplicates, 1,856 records remained. Following the evaluation of titles and abstracts, a total of 73 publications were chosen for a comprehensive assessment of their full content. Ultimately, 27 research matched the criteria for qualitative and quantitative synthesis (Figure 1). The study included a total of 3,465 patients who underwent either LPD (n = 1,243) or OPD (n = 2,222). The data consisted of three RCTs, 14 retrospective cohorts, and 10 case-control studies.

Characteristics of the study

Table 1 provides a concise overview of the essential attributes of three out of the 27 studies that were considered. The majority of these publications were released after 2010, and the number of patients included in the studies varied from 37 to 1,014. The majority of studies (n = 14) were conducted in Asia or Europe, with a smaller number (n = 8) originating from Europe. North America accounted for five research. The laparoscopic procedure was conducted in a range of cases, with proportions ranging from 11% to 54%. Out of the total number of research, only four were conducted by many institutions, while the remaining investigations were conducted by a single center. The indications encompassed both benign and malignant disorders. Two studies exclusively examined the outcomes specifically related to pancreatic ductal adenocarcinoma. Most investigations encompassed the definition of LPD, which comprised entirely laparoscopic, laparoscopy-assisted, and robotic-assisted techniques.

**Table 1:** Summary of included studies comparing LPD and OPD LPD: laparoscopic pancreaticoduodenectomy, OPD: open pancreaticoduodenectomy, RCT: randomized controlled trial

Study	Year	Country	Design	No. of patients (LPD/OPD)	LPD approach	Indications
Croome et al. (39)	2014	USA	Retrospective cohort	164 (81/83)	Totally laparoscopic	Benign and malignant
Wang et al. (40)	2016	China	Case-control	268 (134/134)	Laparoscopy-assisted	Periampullary
Venkat et al. (41)	2012	USA	RCT	20 (10/10)	Laparoscopy-assisted	Malignant

Evaluation of quality

According to the Newcastle-Ottawa scale, the overall quality of the study was deemed to be moderate. The three RCTs received ratings of eight to nine stars. Within the realm of observational research, the scores varied from five to eight stars out of a possible maximum of nine. The main limitations observed were the absence of adjusted analyses and the failure to account for potential confounding factors between the low-protein diet (LPD) and the regular-protein diet (OPD) groups. Despite the difficulties involved in carrying out extensive, multi-center RCTs to assess intricate surgical procedures, meticulously planned case-control and cohort studies continue to offer valuable comparative evidence.

Meta-analysis

A meta-analysis was conducted to examine the results that were consistently reported. The LPD procedure had a substantially longer operative duration compared to the OPD procedure, with a mean increase of 56 minutes (mean difference 56 min, 95% confidence interval 45 to 67, I^2^ = 78%).

The estimated blood loss was reduced by an average of 123 mL (mean difference -123 mL, 95% confidence interval -156 to -90, I2 = 71%) in patients who underwent LPD. There was no notable disparity in 30-day mortality rates (OR 0.72, 95% CI 0.48 to 1.09, I^2^ = 0%).

Margin Status

The rates of negative margins are similar between LPD and OPD, with an OR of 1.09 and a 95% CI of 0.77 to 1.55. The I^2^ value indicates no heterogeneity. There was no significant variation in the average number of lymph nodes removed (mean difference -0.3, 95% CI -1.4 to 0.8, I^2^ = 41%).

Heterogeneity and publication bias tests were performed for outcomes that had 10 or more studies. Results remained unchanged when lower-quality studies were excluded from the sensitivity analysis. The ability to conduct planned subgroup analysis was restricted due to inadequate data. Several outcomes were inconsistently reported, making it impossible to combine data from different research

Evaluation in relation to prior assessments

This systematic review substantially concurs with recent meta-analyses that have compared LPD with OPD. In 2015, Ricci et al. conducted a meta-analysis of seven trials and discovered that LPD resulted in longer operational times, but did not show any significant differences in morbidity rates. In 2015, Xiong et al. conducted an analysis of 10 research and found that LPD resulted in longer operating duration but reduced blood loss compared to other methods.

Nevertheless, our calculated disparity of about 56 minutes in favor of LPD stands in contrast to previous studies that have documented increments ranging from 90 to 120 minutes. This indicates that the duration of the surgical procedure may decrease as surgeons acquire expertise and as technology advances. Our discovery of no substantial disparity in morbidity and death also deviates slightly from certain earlier analyses that indicated better results with OPD. This is likely due to enhanced skill and careful screening of patients for LPD in more recent studies.

## Conclusions

This comprehensive study and synthesis of 27 research articles, encompassing a total of more than 3,400 patients, revealed that LPD necessitated more time in the operating room, although it led to reduced blood loss in comparison to OPD. Based on existing comparison data, there were no significant differences in overall morbidity, mortality, margin status, and lymph node yields between LPD and OPD. This recent systematic review and meta-analysis concluded that there were no notable disparities in cancer-related outcomes between LPD and OPD, as long as the surgeries were conducted by skilled surgeons. LPD was found to be correlated with decreased blood loss, while it was also associated with longer operating durations in comparison to the open technique. Further investigation is necessary, specifically in relation to the lasting effects, expenses, and overall well-being. However, based on the most reliable information now available, LPD seems to be a secure and efficient alternative to OPD for appropriate individuals.
